# The Role of Selected Flavonoids in Modulating Neuroinflammation in Alzheimer’s Disease: Mechanisms and Therapeutic Potential

**DOI:** 10.3390/brainsci15050485

**Published:** 2025-05-05

**Authors:** Joanna Kruszka, Jakub Martyński, Karolina Szewczyk-Golec, Alina Woźniak, Jarosław Nuszkiewicz

**Affiliations:** 1 Student Research Club of Medical Biology and Biochemistry, Department of Medical Biology and Biochemistry, Faculty of Medicine, Ludwik Rydygier Collegium Medicum in Bydgoszcz, Nicolaus Copernicus University in Toruń, 24 Karłowicza St., 85-092 Bydgoszcz, Poland; 310871@stud.umk.pl; 2 Department of Medical Biology and Biochemistry, Faculty of Medicine, Ludwik Rydygier Collegium Medicum in Bydgoszcz, Nicolaus Copernicus University in Toruń, 24 Karłowicza St., 85-092 Bydgoszcz, Poland; kuba.martynski99@gmail.com (J.M.); karosz@cm.umk.pl (K.S.-G.); jnuszkiewicz@cm.umk.pl (J.N.)

**Keywords:** Alzheimer’s disease, amyloid-β, cognitive function, flavonoids, neuroinflammation, neuroprotection, oxidative stress, tau pathology

## Abstract

Alzheimer’s disease (AD) is a progressive neurodegenerative disorder characterized by cognitive decline, amyloid-β (Aβ) deposition, tau hyperphosphorylation, oxidative stress, and chronic neuroinflammation. Growing evidence highlights neuroinflammation—driven by microglial activation and pro-inflammatory cytokine release—as a key contributor to AD pathogenesis and progression. In the absence of effective disease-modifying therapies, attention has turned to natural compounds with multi-target potential. Flavonoids, a diverse class of plant-derived polyphenols, have demonstrated neuroprotective properties through antioxidant activity, modulation of neuroinflammatory pathways, and interference with both Aβ aggregation and tau pathology. This narrative review provides an integrative overview of current findings on the mechanisms of action of key flavonoids—such as quercetin, luteolin, and apigenin—in both preclinical and clinical models. Emphasis is placed on their effects on microglial polarization, oxidative stress reduction, mitochondrial support, and synaptic function enhancement. Moreover, flavonoids show synergistic potential when combined with standard pharmacotherapies, such as acetylcholinesterase inhibitors, and may offer broader cognitive benefits in patients with mild cognitive impairment (MCI). Despite these promising findings, significant challenges persist, including poor bioavailability, inter-individual variability, and limited long-term clinical data. This review identifies critical gaps in knowledge and outlines future directions, including targeted drug delivery systems, biomarker-guided personalization, and long-duration trials. Flavonoids thus emerge not only as promising neuroprotective agents but also as complementary candidates in the development of future multi-modal strategies for AD treatment.

## 1. Introduction

### 1.1. Overview of Alzheimer’s Disease and Neuroinflammation

Alzheimer’s disease (AD), first described by the German psychiatrist and neuropathologist Alois Alzheimer, is a progressive neurodegenerative disorder and the most common cause of dementia worldwide [1]. The first documented case involved a 51-year-old woman who presented with memory loss and personality changes [1]. Histopathological examination revealed characteristic extracellular amyloid-β (Aβ) plaques and intracellular neurofibrillary tangles (NFTs) in the cerebral cortex, along with significant neuronal loss, distinguishing AD from other forms of dementia [2,3]. Currently, approximately 50 million people suffer from AD globally, and this number is projected to rise to 152 million by 2050 due to population aging [4]. The etiology of AD includes both non-modifiable and modifiable risk factors [5]. Non-modifiable risk factors encompass age and genetic predisposition, while modifiable risk factors involve cardiovascular and lifestyle-related factors, such as hypertension, diabetes, obesity, physical inactivity, smoking, low educational attainment, and cognitive inactivity. Notably, the risk of developing AD increases with age, reaching nearly 50% in individuals over 85 years old [6]. Although initial studies failed to demonstrate a strong link between family history and the development of AD, this was likely due to limited knowledge about genetic contributors at the time [7]. Today, several genes have been identified as playing crucial roles, especially in early-onset familial AD. These include the amyloid precursor protein (*APP*) gene, presenilin 1 and 2 (*PSEN1*, *PSEN2*), and genes involved in lipid metabolism and cholesterol transport, such as apolipoprotein E (*APOE*), apolipoprotein C1 (*APOC1*), and apolipoprotein J (*APOJ*) [2]. Among the key pathological processes driving AD progression, neuroinflammation has gained significant attention [8]. Over the past two decades, studies have increasingly emphasized the role of the brain’s innate immune system, particularly microglia, in the initiation and propagation of AD pathology. In AD, microglia become chronically activated, releasing pro-inflammatory cytokines and other neurotoxic factors that exacerbate neuronal injury [9]. Persistent neuroinflammation not only contributes to neuronal death but also promotes the accumulation of Aβ plaques and NFTs, hallmark features of AD [10]. Furthermore, the deposition of Aβ plaques is believed to trigger a chronic inflammatory response. Activated microglia, while initially attempting to clear amyloid deposits, can become overactive and enhance tau pathology, thereby disrupting normal neuronal function and exacerbating synaptic dysfunction [11].

Oxidative stress is a condition characterized by an imbalance between the production of reactive oxygen species (ROS) and the cells’ ability to neutralize them [12]. As a result, the body experiences the detrimental effects of free radicals, also known as ROS, which damage cells and tissues. ROS are molecules containing an O–O bond or oxygen atoms with unpaired electrons, including hydroxyl radicals (^•^OH), superoxide anions (O_2_^•−^), singlet oxygen (^1^O_2_), and nitrogen-derived free radicals, such as nitric oxide (NO) and peroxynitrite (ONOO^−^) [13]. At low concentrations, ROS play physiological roles, such as participating in muscle contractions and exerting bactericidal effects [13]. However, in excessive amounts, ROS become highly reactive and harmful because they aggressively bind to other molecules to stabilize their electron configuration, potentially leading to the formation of unstable and damaging compounds [14]. ROS are naturally generated during normal metabolic processes, primarily in mitochondrial respiration, where mitochondria produce energy in the form of adenosine triphosphate (ATP) [15]. Oxidative stress can damage proteins and nucleic acids, impair enzymatic activity, and disrupt overall cellular function. Although cells possess mechanisms to counteract minor oxidative damage and restore homeostasis, excessive oxidative stress can induce apoptosis and, in severe cases, necrosis [16]. Furthermore, oxidative stress plays a crucial role in the pathogenesis of numerous diseases, including cancer, Parkinson’s disease (PD), myocardial infarction, and AD. ROS-induced lipid peroxidation leads to the generation of lipid radicals, which compromise the structural integrity of cell membranes. Additionally, oxidative damage to DNA and RNA can result in genetic mutations and impaired cellular function [17]. The brain is particularly susceptible to oxidative damage due to its exceptionally high oxygen consumption, abundant lipid content, and relatively low levels of endogenous antioxidant defenses. Neuronal membranes are rich in polyunsaturated fatty acids, which are highly vulnerable to lipid peroxidation by ROS. Additionally, the brain’s limited capacity to regenerate damaged cells and its reliance on mitochondrial energy production further increase its sensitivity to oxidative stress. These factors collectively make the brain highly prone to oxidative injury, which plays a critical role in the pathogenesis of neurodegenerative diseases [14,16,17].

### 1.2. Flavonoids as Emerging Therapeutics

Flavonoids are a diverse group of plant-derived compounds widely recognized for their potent antioxidant and anti-inflammatory properties [18]. These bioactive molecules are abundant in various foods, including fruits, vegetables, tea (*Camellia sinensis*), and cocoa (*Theobroma cacao*) [19]. Flavonoids exert antioxidant effects primarily through the scavenging of ROS, thereby reducing oxidative stress that can otherwise damage cellular components and tissues [20]. In addition to their antioxidant action, flavonoids modulate inflammatory pathways by attenuating the production of pro-inflammatory cytokines and influencing immune cell activity [21].

In the context of AD, a neurodegenerative disorder characterized by chronic neuroinflammation and oxidative stress, flavonoids have demonstrated promising neuroprotective effects. Both oxidative stress and neuroinflammation are critical factors that contribute to AD progression, leading to neuronal injury, synaptic dysfunction, and ultimately cognitive decline [22]. Flavonoids mitigate these pathological processes by reducing oxidative damage to neurons and glial cells and by suppressing neuroinflammatory pathways, including the activation of microglia and the release of pro-inflammatory cytokines such as tumor necrosis factor-alpha (TNF-α) and interleukin-6 (IL-6) [23]. Moreover, several studies suggest that flavonoids may enhance mitochondrial function, promote the clearance of Aβ plaques, and improve synaptic plasticity, thereby offering a multifaceted neuroprotective approach in AD [22,24].

The aim of this review is to examine the potential of selected flavonoids to modulate neuroinflammatory mechanisms involved in AD and to explore their therapeutic implications. We focused on selected flavonoids that have been most extensively studied in preclinical and clinical models of AD. These compounds not only represent diverse structural subclasses but also provide sufficient mechanistic and translational data to support a focused, integrative analysis. To construct this narrative review, we conducted a comprehensive literature search in the PubMed, Google Scholar, and Web of Science databases, focusing on articles published within the past 10 to 15 years. The search terms included “Alzheimer’s disease”, “neuroinflammation”, “flavonoids”, “oxidative stress”, “microglia”, “amyloid-β”, and “tau pathology”. Only articles published in English or Polish were considered. Inclusion criteria encompassed original research articles, reviews, and meta-analyses investigating the role of flavonoids in neuroinflammation, oxidative stress, or AD pathophysiology. Studies were excluded if they were unrelated to neurodegenerative processes, lacked mechanistic discussion, or were not peer-reviewed. Additionally, reference lists of selected papers were manually screened for further relevant studies. Emphasis was placed on preclinical studies, clinical trials, and experimental models to provide a comprehensive synthesis of recent findings. Unlike previous reviews, which often focus narrowly on oxidative stress or amyloid pathology alone, this article highlights the dual role of flavonoids in modulating both neuroinflammation and oxidative stress, as well as their impact on Aβ and tau pathology, offering a more integrative therapeutic perspective in AD. Moreover, the review underscores emerging evidence suggesting that flavonoids not only interfere with pathological processes but may also modulate microglial polarization and support synaptic function—aspects that remain underexplored in current AD research.

Despite these promising findings, significant gaps in knowledge persist. A major limitation is the poor bioavailability of flavonoids, which severely restricts their therapeutic application in clinical settings. Although novel delivery methods, such as nanoformulations, are being explored to enhance their absorption and stability, human clinical trials utilizing these technologies remain scarce. Additionally, there is a lack of long-term, large-scale clinical trials assessing the cognitive effects of flavonoid supplementation in diverse patient populations. The specific molecular mechanisms by which individual flavonoids influence microglial activation, mitochondrial function, and synaptic plasticity in the human AD brain are also not fully elucidated. Finally, the potential synergistic effects of flavonoids combined with existing pharmacotherapies, such as acetylcholinesterase inhibitors, represent a field requiring further investigation to optimize combination treatment strategies. By addressing these knowledge gaps and synthesizing current data, this review aims to identify future research directions that could facilitate the translation of flavonoid-based therapeutic strategies into clinical practice.

## 2. Neuroinflammation in Alzheimer’s Disease

### 2.1. Neuroinflammatory Mechanisms in AD

In AD, microglia adopt a pro-inflammatory phenotype and release cytokines and ROS that contribute to disease progression. Their role in neuroinflammation is further detailed in the following sections [25].

Microglia act as the brain’s innate immune cells, serving as the first line of defense against injury and disease. Under physiological conditions, they maintain homeostasis by clearing debris, dead cells, and pathogens [26]. However, in AD, microglia become chronically activated in response to the accumulation of Aβ plaques in the extracellular space and tau tangles within neurons. Microglia recognize Aβ deposits and tau aggregates as harmful, triggering their activation. While initially aimed at clearing Aβ and damaged cellular components, microglia in AD become overactive, releasing excessive pro-inflammatory mediators and ROS, thus sustaining chronic inflammation [27].

Activated microglia produce ROS as part of their immune response to Aβ and tau. These reactive molecules damage neuronal membranes, proteins, and DNA, and their accumulation interferes with mitochondrial function, contributing to neuronal dysfunction and death [28,29]. Moreover, ROS further enhances microglial activation, creating a self-perpetuating feedback loop of neuroinflammation and oxidative stress [29].

Upon activation, microglia release a wide range of pro-inflammatory cytokines, including IL-1β, IL-6, and TNF-α, that play crucial roles in propagating neuroinflammation and exacerbating neuronal injury [30]. IL-1β is one of the most potent pro-inflammatory cytokines secreted by microglia, promoting the expression of other inflammatory mediators, increasing blood–brain barrier (BBB) permeability, and contributing to neuronal excitotoxicity. Furthermore, IL-1β exacerbates Aβ accumulation and induces tau hyperphosphorylation, amplifying neuronal and synaptic dysfunction [31].

IL-6 contributes both to the initiation and maintenance of neuroinflammation. Its elevated levels are associated with neurodegeneration and cognitive decline in AD [32]. IL-6 also promotes the activation of astrocytes, another key glial cell type, which in turn release additional pro-inflammatory mediators and contribute to synaptic dysfunction. Importantly, IL-6 disrupts synaptic plasticity, impairing memory formation and learning processes [33].

TNF-α, produced by activated microglia and astrocytes, is a major effector of neuroinflammation [34]. It induces neuronal apoptosis, enhances oxidative stress, and promotes the aggregation of Aβ, creating a vicious cycle of inflammation and neurodegeneration [33].

Astrocytes, alongside microglia, play a synergistic role in sustaining neuroinflammation. Once activated, astrocytes contribute to cytokine release, impair glutamate uptake, and secrete NO and ROS, exacerbating excitotoxic damage to neurons [35]. Furthermore, astrocytic dysfunction in AD leads to impaired clearance of extracellular glutamate, promoting glutamate spillover, which overstimulates glutamate receptors on neurons, including N-methyl-D-aspartate (NMDA) receptors, resulting in calcium influx, mitochondrial damage, and apoptosis [9,36].

The prolonged release of pro-inflammatory cytokines and sustained oxidative stress lead to chronic excitotoxicity, which accelerates the loss of neurons, especially in brain regions crucial for memory and cognition, such as the hippocampus and cortex [36]. Additionally, chronic inflammation and oxidative stress impair neurogenesis, particularly within the hippocampus, further limiting the brain’s capacity for repair and contributing to cognitive decline [37].

### 2.2. The Feedback Loop: Oxidative Stress and Inflammation

Aβ plaques and tau tangles directly promote the generation of ROS, which are critical contributors to neuronal injury in AD [38]. In an attempt to clear Aβ, microglia become activated and release ROS. Although this response is initially protective, prolonged microglial activation leads to excessive production of ROS and pro-inflammatory cytokines, exacerbating neuronal damage [39].

Activated microglia sustain inflammatory signaling through the release of cytokines and ROS, amplifying neuronal damage in AD [40]. This interaction creates a vicious cycle in which inflammation promotes oxidative damage, which in turn amplifies inflammation, leading to chronic neuroinflammation and oxidative stress [41].

Furthermore, oxidative stress and inflammatory cytokines disrupt synaptic plasticity, a key process underpinning learning and memory [35]. ROS and pro-inflammatory mediators damage synaptic proteins, alter intracellular signaling pathways that regulate synaptic strength and impair neuronal communication. As a result, synaptic dysfunction develops, impeding memory formation and cognitive processes [42].

The continuous activation of microglia, persistent cytokine release, and sustained oxidative stress establish a self-perpetuating loop that accelerates AD progression [43]. Damage inflicted on neurons through chronic inflammation and ROS generation promotes further accumulation of Aβ and tau aggregates, which subsequently activates additional microglia, amplifying cytokine production and ROS release. This destructive cycle drives the progressive loss of neurons and synapses, ultimately leading to the cognitive decline characteristic of AD [39,44].

Mitochondria, essential for neuronal energy metabolism, are especially vulnerable to oxidative stress. ROS-induced mitochondrial damage disrupts ATP production, resulting in energy deficits that impair neuronal and synaptic function [45]. Moreover, dysfunctional mitochondria become additional sources of ROS, perpetuating the cycle of oxidative stress and inflammation [46]. The combined impact of chronic oxidative stress and inflammation not only leads to progressive synaptic failure but also triggers apoptotic pathways. Consequently, neuronal death, particularly in brain regions critical for memory and cognition such as the hippocampus and cortex, underlies the severe cognitive deficits observed in AD [47]. The most important inflammatory markers involved in AD pathophysiology are summarized in Table 1.

## 3. Flavonoids: Overview and Classification

### 3.1. Chemical Structure and Subclasses of Flavonoids

Flavonoids are a diverse group of organic compounds with a characteristic polyphenolic structure. They belong to secondary plant metabolites and are widely distributed throughout the plant kingdom [54]. In plants, flavonoids play essential biological roles, including protection against ultraviolet (UV) radiation, oxidative stress, pathogens, and various environmental stressors [55]. They also function as signaling molecules and contribute to the color, taste, and fragrance of flowers and fruits, thereby attracting pollinators and animals that aid in seed dispersal. Flavonoids are found in all parts of the plant, including leaves, stems, roots, and reproductive organs [19,56].

Beyond their role in plants, flavonoids exhibit a broad range of therapeutic properties in animals and humans. Due to their antioxidant, anti-inflammatory, anti-mutagenic, anti-carcinogenic, and neuroprotective activities, they are utilized in nutritional, pharmaceutical, medical, and cosmetic applications [57]. Flavonoids have been studied for their beneficial effects in cardiovascular diseases, cancer, atherosclerosis, and neurodegenerative disorders such as AD. They are also known to modulate the activity of key cellular enzymes involved in metabolism and signal transduction [19,58].

The basic structure of flavonoids is the 2-phenylchroman (or C6–C3–C6) backbone, consisting of two aromatic rings (A and B) connected by a heterocyclic pyran ring (C) [59]. Structural diversity among flavonoids arises from the number and position of hydroxyl, methoxyl, or glycosidic substituents, the degree of hydroxylation, and polymerization. These variations influence their chemical properties, metabolic pathways, and biological activity [58].

To date, more than 9000 different flavonoid compounds have been identified and classified into several major subclasses, including chalcones, flavones, flavonols, flavanones, flavans, biflavonoids, isoflavones, and anthocyanidins [19]. Among these, flavonols, flavones, and isoflavones (particularly from soy (*Glycine max*)) are the most abundant in the human diet [60].

The antioxidant activity of flavonoids is attributed to several mechanisms, including the inhibition of oxidative enzymes and the direct scavenging of ROS [61]. In many cases, flavonoids act as radical-trapping agents. Their antioxidant potential is closely linked to structural features, particularly the presence of hydroxyl groups capable of donating electrons and stabilizing the resulting flavonoid radical via delocalization across the aromatic system [20].

### 3.2. Selected Subclasses of Flavonoids

#### 3.2.1. Flavonols

Flavonols are a subclass of flavonoids derived from 3-hydroxyflavone (according to IUPAC nomenclature: 3-hydroxy-2-phenylchromen-4-one), which constitutes their central structure. A distinguishing feature of this group is the presence of a hydroxyl group at the C3 position of the C ring, which differentiates flavonols from flavones. For this reason, they are sometimes referred to as hydroxy derivatives of flavones [62].

The structural diversity of flavonols is primarily determined by the number and position of hydroxyl or methyl substituents, which influence their chemical reactivity, stability, and antioxidant properties. In general, flavonols with fewer hydroxyl groups are more chemically stable, whereas those with more –OH groups exhibit higher reactivity and stronger antioxidant activity [63].

Flavonols are abundant in many plant-based foods, especially vegetables such as onion (*Allium cepa*), lettuce (*Lactuca sativa*), tomatoes *(Solanum lycopersicum* L.), spinach (*Spinacia oleracea*), and cauliflower (*Brassica oleracea*), as well as fruits like strawberries (*Fragaria ananassa*), apples (*Malus pumila*), grapes (*Vitis vinifera*), and blueberries (*Vaccinium angustifolium*). They are also present in medicinal plants, including *Moringa oleifera* leaves, *Aloe vera* leaves, and *Ficus religiosa* fruits. Additional sources include wine and green tea (*Camellia sinensis*) [19,64].

In plant tissues, flavonols are most commonly found not as free compounds (aglycones) but in the form of glycosides. These are molecules in which one or more sugar residues are covalently bonded to the phenolic hydroxyl group via glycosidic bonds [65]. The most frequent forms in food are *O*-*β*-glycosides, where the sugar moiety is linked to the oxygen atom of the flavonol structure [66].

Among all flavonols, quercetin is one of the most common and biologically potent representatives. It is known for its strong antioxidant capacity, but it also exhibits anti-inflammatory, antiviral, anticancer, and neurostimulatory effects [67]. Additionally, quercetin has been shown to stimulate mitochondrial biogenesis and inhibit platelet aggregation, further broadening its physiological impact. Dietary intake of quercetin varies by geographical region and is influenced by individual consumption of fruits, vegetables, and tea (*Camellia sinensis*) [68].

#### 3.2.2. Flavones

Flavones constitute one of the largest subclasses of flavonoids and perform diverse biological functions in plants. They act as pigments, UVB filters, natural pesticides, and signaling molecules involved in plant defense mechanisms and communication [69]. In addition to their role in plants, flavones exhibit multiple health-promoting effects in humans, including antioxidant, anti-inflammatory, anticancer, and cardioprotective properties [70].

Structurally, flavones are based on the 2-phenyl-1-benzopyran-4-one skeleton. They are distinguished from other flavonoids by the presence of a double bond between carbon atoms C2 and C3 in the C ring and a carbonyl group at the C4 position. Typically, a hydroxyl group is present at the C5 position of the A ring, and in many compounds, it is also present at C7 of the A ring or C3′ and C4′ positions of the B ring. Notably, the C3 position is unsubstituted, which differentiates flavones from flavonols [69,71].

Like flavonols, flavones are commonly found in plants in the form of glycosides, where sugar residues are attached to hydroxyl groups via glycosidic bonds. Dietary sources of flavones include celery (*Apium graveolens*), parsley (*Petroselinum crispum*), and chamomile (*Matricaria chamomilla* and *Chamaemelum nobile*) [19], as well as grape (*Vitis vinifera*) skin and wine [70], A particularly rich source of flavones is found in citrus fruits, especially bergamot (*Citrus Bergamia*) [72].

Frequently studied flavones include luteolin, apigenin, tangeretin, and nobiletin—compounds known for their anti-inflammatory, neuroprotective, and anti-cancer potential [19,73].

#### 3.2.3. Flavanones

Flavanones (also known as dihydroflavones) differ structurally from flavones and flavonols by the absence of a double bond between the C2 and C3 positions in the C ring, which results in a saturated (non-planar) heterocyclic ring structure [71]. This saturated configuration gives flavanones distinct chemical properties compared to other flavonoid subclasses [74].

Unlike other flavonoids, flavanones have a relatively restricted natural distribution, being found primarily in citrus fruits, tomatoes *(Solanum lycopersicum* L.), and selected aromatic herbs such as mint (*Mentha* spp.). Despite this limited botanical occurrence, flavanones constitute a significant portion of flavonoid intake in the human diet due to the widespread consumption of citrus fruits and fruit juices [75]. In Europe, oranges (*Citrus sinensis*) and orange juice are the predominant dietary sources of flavanones [76].

Prominent representatives of this subclass include naringenin and hesperidin, both of which contribute to the characteristic bitter flavor of citrus fruits such as grapefruits (*Citrus paradisi*) [77]. In addition to their sensory properties, flavanones possess a variety of biological activities, including antioxidant, anti-inflammatory, and anti-carcinogenic effects. These functions are linked to their ability to modulate enzyme activity, inhibit oxidative damage, and influence cellular signaling pathways relevant to inflammation and cancer development [78].

#### 3.2.4. Isoflavones

A characteristic structural feature of isoflavones is the position of the B ring, which is attached to the C3 position of the central C ring, rather than the C2 position as seen in most other flavonoids [71]. This unique configuration gives isoflavones distinct biological properties. Isoflavones are secondary plant metabolites primarily synthesized as part of the plant’s defense system, particularly for their antifungal activity.

They play a particularly important role in leguminous plants, where they contribute to establishing symbiotic interactions with rhizobial bacteria, resulting in the formation of nitrogen-fixing root nodules. The concentration of isoflavones in plants depends on environmental factors such as temperature, humidity, soil fertility, and exposure to pathogens [79,80]. Isoflavones occur in several chemical forms, including aglycones, *7*-*O*-glucosides, *6*″-*O*-acetyl-*7*-*O*-glucosides, and *6*″-*O*-malonyl-*7*-*O*-glucosides [81]. In plants, they are predominantly present as inactive glycosides, which are enzymatically hydrolyzed in the human intestine to their bioactive aglycone forms. Fermentation processes, such as those used in the production of tempeh or miso, have been shown to enhance the bioavailability of isoflavones by increasing the proportion of aglycones [82].

Due to their structural similarity to 17*β*-estradiol, isoflavones can bind selectively to estrogen receptors α and β, exhibiting estrogen-like (phytoestrogenic) activity [83]. This property has attracted attention in the context of hormone-dependent conditions.

Isoflavones have demonstrated a range of health-promoting effects, including anti-diabetic and anti-obesity actions, as well as anti-cancer properties. They also inhibit bone resorption, reducing the risk of osteoporosis [84,85], and may contribute to neuroprotection, potentially lowering the risk of developing neurodegenerative diseases such as AD [86].

#### 3.2.5. Flavanols (Flavan-3-Ols)

Flavanols, also known as flavan-3-ols, dihydroflavonols, or catechins, form a structurally distinct subclass of flavonoids. Unlike flavones and flavonols, flavanols lack both a double bond between the C2 and C3 positions and a carbonyl group at the C4 position. Instead, they possess a hydroxyl group at the C3 position of the C ring, which introduces two chiral centers into the molecule and contributes to their stereochemical diversity [19,71].

Flavanols are considered a highly heterogeneous group and are found in monomeric (e.g., catechin, epicatechin), oligomeric (procyanidins), and polymeric (condensed tannins) forms. Unlike many other flavonoids, they are not commonly glycosylated in food sources [87].

Dietary sources of flavanols include tea (*Camellia sinensis*) (particularly green and black varieties), red wine, grapes (*Vitis vinifera*), apples (*Malus pumila*), cocoa (*Theobroma cacao*), and cocoa-derived products, especially dark chocolate, which is particularly rich in flavanol monomers and oligomers [88,89].

One of the most studied biological actions of flavanols is their ability to increase NO bioavailability in the vascular endothelium. This leads to vasodilation, improved endothelial function, and a subsequent reduction in blood pressure [90]. These effects are particularly significant in the context of cardiovascular disease prevention.

Owing to their potent antioxidant and anti-inflammatory properties, flavanols may play a protective role in the prevention of several chronic conditions, including cardiovascular diseases, diabetes, and neurodegenerative disorders such as AD and PD [91].

#### 3.2.6. Anthocyanins

Anthocyanins are a group of water-soluble flavonoid pigments that serve as secondary metabolites in plants, responsible for the red, purple, and blue coloration of flowers, fruits, and leaves [92]. These compounds play important roles in attracting pollinators, protecting against UV radiation, and defending against pathogens.

The term anthocyanins refers to the glycosylated forms of these pigments, while their aglycone (non-glycosylated) counterparts are called anthocyanidins. The core structure of anthocyanidins is based on the 2-phenylbenzopyrylium (flavylium) ion, which is responsible for their color and reactivity. Due to their chemical instability and high reactivity, anthocyanidins rarely occur in free form in nature; glycosylation increases their solubility and stability, especially in aqueous environments [93].

In most anthocyanins, glycosylation occurs at the C3 position of the C ring, although additional sugar residues may be attached at C5 or C7 positions, depending on the compound [94]. In most anthocyanins, glycosylation occurs at the C3 position of the C ring, although additional sugar residues may be attached at C5 or C7 positions, depending on the compound [95].

To date, over 500 distinct anthocyanins have been isolated from plant sources [96]. Numerous studies have demonstrated that anthocyanins possess a broad spectrum of health-promoting effects. These include antioxidant, anti-cancer, anti-mutagenic, anti-proliferative, antimicrobial, antiviral, anti-inflammatory, and anti-allergic activities [97].

Anthocyanin-rich foods include blueberries (*Vaccinium angustifolium*), black elderberries (*Sambucus nigra*), mulberries (*Morus australis* P.), blackcurrants (*Ribes nigrum*), cherries (*Prunus avium* L.), black soybeans (*Glycine max* (L.) *Merr.*), and red cabbage microgreens (*Brassica oleracea* L. var. *capitata*) [98]. Their health benefits, combined with their natural origin and safety, make anthocyanins attractive candidates for both functional food development and nutraceutical applications.

### 3.3. Bioavailability and Dietary Sources

Flavonoids are widespread in nature and are found in a variety of plant-based foods. Major dietary sources include fruits, vegetables, wine [56], black and green tea (*Camellia sinensis*) [58], cocoa (*Theobroma cacao*) powder, and chocolate [59,89], as well as various herbs and medicinal plants [19].

Following oral administration, flavonoids are absorbed predominantly in the small intestine, to a lesser extent in the large intestine, and minimally in the stomach [99,100]. Their bioavailability depends on several factors, most notably their chemical structure. Key determinants include the presence of free hydroxyl groups, the degree of glycosylation, the type of sugar moiety, and the potential for rapid conjugation reactions, such as glucuronidation and sulfation [101].

In Caco-2 cell models, flavonoid uptake is influenced by the molecule’s hydrophilicity or hydrophobicity. The presence of hydrophilic sugar moieties can hinder the absorption of flavonoid glycosides by reducing their ability to cross cellular membranes [102]. However, this relationship has been clearly demonstrated in vivo only for certain compounds. For instance, quercetin-*3*-*O*-glucoside is absorbed more efficiently than quercetin-*3*-*O*-rhamnoside, likely due to the role of the intestinal microbiota [102].

Gut microbiota are responsible for hydrolyzing glycosylated flavonoids into aglycones, which can then be absorbed by passive diffusion through the intestinal epithelium. This makes the intestinal microbiome a crucial determinant of flavonoid absorption and metabolism [100].

In addition, other dietary components also influence flavonoid uptake. Their absorption is enhanced in the presence of dietary fats and reduced when consumed with proteins [103]. pH conditions are also important: studies using Caco-2 cells suggest that a slightly acidic to neutral environment enhances the permeability of certain flavonoids, particularly quercetin [104].

The liver is the primary site of flavonoid metabolism, where these compounds undergo two major types of biotransformation reactions: oxidation and conjugation [105]. Oxidative metabolism is catalyzed predominantly by cytochrome P450 enzymes, which introduce polar functional groups to the molecule, increasing its solubility. Conjugation involves the activity of phase II enzymes, such as UDP-glucuronosyltransferases, sulfotransferases, and catechol-*O*-methyltransferases, which facilitate the binding of flavonoids to endogenous polar compounds like glucuronic acid, sulfate groups, or methyl groups [106].

Due to their often limited bioavailability, improving the absorption and stability of flavonoids remains a key challenge and has become the focus of various technological approaches. Among the most promising strategies is the use of nanotechnology-based delivery systems, including liposomes, nanoemulsions, and nanoencapsulation [107].

Liposomes, composed of phospholipid bilayers, are able to integrate into biological membranes, allowing for efficient transport of lipophilic (non-polar) molecules across tissues. Their encapsulation of flavonoids such as naringin has been shown to increase antiproliferative activity and significantly improve oral bioavailability [108,109].

Another approach is the use of nanoemulsions, which help stabilize flavonoids in aqueous environments and enhance their absorption. Studies involving quercetin and gallic acid nanoemulsions have demonstrated increased water solubility and reduced chemical degradation [110].

Nanoencapsulation, on the other hand, refers to enclosing functional compounds within nanoscale vesicles or carriers, which can protect flavonoids from environmental factors such as heat, oxidation, or enzymatic hydrolysis. For example, quercetin encapsulated in polylactide (PLA) nanocarriers exhibited improved solubility and enhanced physicochemical stability [104].

These advanced formulations protect flavonoids not only after ingestion but also during pre-consumption stages, such as storage or thermal processing of flavonoid-rich foods. As a result, nanotechnology offers significant potential for enhancing the efficacy and shelf-life of flavonoid-based supplements and functional foods [106].

Numerous studies have demonstrated that flavonoids interact with other food components, such as proteins, carbohydrates, and lipids [110]. These interactions can either enhance or reduce flavonoid bioavailability, depending on the chemical structure of the flavonoid and the nature of the interacting compound [110].

Among these, protein–flavonoid interactions are the most extensively studied [111]. Recent research has focused on exploiting these interactions to increase the antioxidant capacity, improve the delivery, and enhance the stability of flavonoids. For example, albumin has been shown to protect flavonoids from degradation during storage by reducing their accessibility to oxidative and enzymatic reactions [112].

Proteins are also employed in encapsulation strategies. A notable example is quercetin encapsulated in zein–sodium caseinate nanoparticles, which provides enhanced protection against degradation and improves molecular stability. Such protein-based carriers can also affect the size, shape, and conformation of flavonoid–protein complexes, as well as influence polymer interactions and aggregation behavior [113,114].

With respect to carbohydrates, flavonoids may interact with both digestible forms (such as starch) and indigestible forms (such as dietary fiber) [115]. Depending on the type of starch and the specific flavonoid, these interactions can produce contradictory effects—either increasing or decreasing starch digestibility, which in turn alters the nutritional and functional properties of food products. Some flavonoid–starch combinations may also induce structural modifications in starch, affecting its enzyme accessibility and breakdown [116]. In the case of dietary fiber, it has been shown to act as a carrier of polyphenols, aiding in their delivery. Additionally, non-starch polysaccharides may either stabilize or interfere with flavonoid binding to proteins, further influencing their availability [117,118].

Lipids also play a key role in the absorption of flavonoids, particularly through lipophilic transport pathways. For instance, the absorption of quercetin has been shown to improve in the presence of dietary fats, likely due to increased solubility and micelle formation [119]. Moreover, flavonoids such as quercetin may bind to lipoprotein fractions, including high-density lipoproteins (HDL), which could further influence their distribution and bioactivity in vivo [120].

## 4. Neuroprotective and Anti-Inflammatory Properties of Flavonoids

### 4.1. Antioxidant Mechanisms of Flavonoids

**Figure 1:**
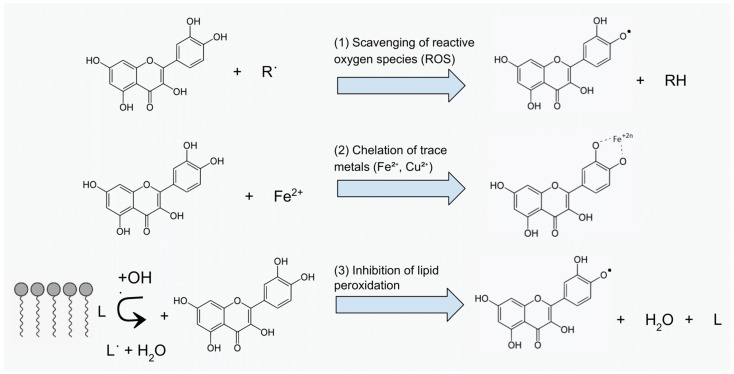
Antioxidant mechanisms of flavonoids: (1) ROS scavenging; (2) metal chelation; (3) inhibition of lipid peroxidation.

Flavonoids exhibit antioxidant activity through several key mechanisms, including scavenging ROS, chelating trace metals, and inhibiting lipid peroxidation (Figure 1). The effectiveness of these actions depends largely on the chemical structure of the flavonoid and the type of ROS involved—flavonoids display the greatest reactivity toward hydroxyl radicals [121].

The ROS-scavenging process is enabled by the presence of hydroxyl groups on the B ring, in conjunction with a C2=C3 double bond conjugated with a C4 carbonyl group [121]. These structural features promote electron delocalization, which is essential for stabilizing the resulting flavonoid radical. Upon reaction with ROS, the flavonoid donates a hydrogen atom and an electron, forming a radical species that can undergo one of three fates: (1) conjugation with another radical; (2) donation of an additional hydrogen, forming a quinone; or (3) dimerization with another flavonoid radical. The stability of the resulting radical determines its reaction pathway [121].

Importantly, compounds containing a catechol moiety (ortho-dihydroxy substitution) in the B ring exhibit the highest antioxidant potential, even more so than flavonoids with a greater number of hydroxyl groups overall [122]. Substitutions such as glycosylation and methylation reduce antioxidant capacity due to steric hindrance and impaired electron delocalization. Flavonoids with only a single hydroxyl group also show reduced activity [123,124].

Another crucial antioxidant mechanism is metal ion chelation. Flavonoids can bind transition metals (e.g., Fe^2+^, Cu^2+^), especially via the 3-hydroxyl and 4-carbonyl groups in the C ring, typically in a 2:1 stoichiometric ratio [125]. This is important because iron catalyzes hydroxyl radical formation via the Fenton reaction. The presence of a catechol group at positions 3′ and 4′ on the B ring, a 5-hydroxyl group on the A ring, and a 2,3 double bond further enhance chelation [126]. Among these, the 5-OH/4-oxo site is considered the most favorable due to the stability of the chelate ring. The number and position of hydroxyl groups ultimately determine chelating capacity [125,126].

Since iron and copper also catalyze lipid peroxidation by generating lipid radicals, their chelation by flavonoids further enhances the overall antioxidant effect [127].

Among individual compounds, quercetin is particularly notable due to its catechol B ring and 3-OH group on the C ring, which confer strong antioxidant properties [128]. In addition, quercetin induces Nrf2–ARE signaling and the expression of paraoxonase 2 (PON2), both of which contribute to cellular defense against oxidative stress [129]. Apigenin has also been shown to enhance endogenous antioxidant systems by increasing glutathione (GSH) levels and demonstrates strong peroxide radical scavenging in the oxygen radical absorbance capacity (ORAC) assay, which is based on the hydrogen atom transfer (HAT) mechanism [67,130].

### 4.2. Anti-Inflammatory Properties of Flavonoids

Flavonoids exert anti-inflammatory effects through multiple mechanisms, including the modulation of enzyme activity (e.g., protein kinases, phosphodiesterases), inhibition of arachidonic acid metabolism (via phospholipase A2 [PLA2], cyclooxygenase [COX], and lipoxygenase [LOX]), and direct interaction with immune cells [131]. They play a significant role in the regulation of inflammatory mediators, such as cytokines, chemokines, and NO [131].

A key target of flavonoid activity is nuclear factor kappa B (NF-κB), a transcription factor responsible for the expression of various pro-inflammatory genes, including those encoding TNF-α, IL-1β, IL-6, and interleukin-8 (IL-8) [132]. Under inflammatory conditions, the endogenous inhibitor of NF-kB (IκB) becomes phosphorylated and subsequently degraded, allowing NF-κB to translocate into the nucleus and initiate transcription of pro-inflammatory genes [132,133]. Although the precise molecular details of this pathway are still under investigation, it represents a promising therapeutic target in numerous chronic inflammatory diseases.

Flavonoids have been shown to modulate this pathway by stabilizing IκB and inhibiting NF-κB nuclear translocation. Moreover, they can influence CD4^+^ T helper 2 (Th2) cell responses by regulating transcriptional factors such as GATA binding protein 3 (GATA-3) and activator of transcription 6 (STAT-6), thereby altering cytokine expression profiles [134,135].

In in vitro studies, quercetin demonstrated potent anti-inflammatory activity by inhibiting COX-2, LOX, and inducible nitric oxide synthase (iNOS), as well as by reducing the production of IL-1β and IL-6. It also suppressed the expression of the p65 subunit of NF-κB, indicating direct inhibition of transcriptional activation [136,137].

Furthermore, in vivo studies in rat models confirmed the anti-inflammatory effects of quercetin. Specifically, it led to reduced levels of TNF-α and NO in adipose tissue and downregulated the expression of nitric oxide synthase (NOS), supporting its therapeutic potential in inflammation-related conditions [68].

## 5. Flavonoids in Modulating Neuroinflammation in Alzheimer’s Disease

### 5.1. Effect on Microglial Activation and Neuroinflammation

Under physiological conditions, microglial cells play a key role in maintaining central nervous system (CNS) homeostasis by clearing cellular debris, modulating synaptic activity, and responding to injury or disease [26]. However, under pathological stimuli, such as Aβ accumulation or exposure to endotoxins, microglia may become chronically activated. This activation results in the release of pro-inflammatory cytokines, ROS, and other neurotoxic mediators, contributing to sustained neuroinflammation and neuronal dysfunction [138].

Among the flavonoids studied for their ability to modulate microglial activation, luteolin has received considerable attention. This compound, found in celery, parsley, and chamomile, has demonstrated anti-inflammatory and neuroprotective properties in multiple preclinical studies [139]. Luteolin was shown to inhibit NF-κB activation and reduce pro-inflammatory cytokine release, leading to cognitive improvement in 3 × Tg-AD transgenic mice. In microglial cultures, luteolin attenuated lipopolysaccharide (LPS)-induced production of TNF-α and IL-6, an effect associated with suppression of the NF-κB signaling pathway, a central regulator of neuroinflammation [140,141]. Furthermore, luteolin’s antioxidant properties contribute to the mitigation of oxidative stress in microglia. For example, a study by Xiong et al. [142] demonstrated that luteolin reduced ROS levels and protected against LPS-induced neuroinflammation in mice.

Quercetin, another well-studied flavonoid abundant in apples (*Malus pumila*), onions (*Allium cepa*), and berries, similarly modulates microglial activation through anti-inflammatory, antioxidant, and mitochondria-targeted mechanisms [143]. In an experimental model, Han et al. [144] reported that quercetin significantly reduced LPS-induced inflammatory cytokine production, microglial proliferation, and NF-κB activation. Additionally, quercetin promoted mitophagy, enhancing the clearance of damaged mitochondria and reducing mitochondrial ROS (mtROS) accumulation. As a result, quercetin treatment protected primary neurons from LPS-induced microglial toxicity and alleviated neurodegeneration in mice.

Further supporting evidence comes from Tsai et al. [145], who showed that quercetin inhibited the expression of lipocalin-2 in both macrophages and microglia stimulated with LPS. Quercetin also reduced NO production and expression of pro-inflammatory cytokines, including IL-6, TNF-α, and IL-1β. Moreover, it significantly reduced intracellular ROS levels in microglia and upregulated IL-10 expression and other endogenous antioxidants, suggesting a shift toward an M2 anti-inflammatory phenotype.

### 5.2. Influence on Amyloid-β and Tau Pathology

The aggregation of Aβ peptides and the formation of extracellular plaques are hallmark features of AD. These pathological deposits are closely associated with neuronal injury, synaptic dysfunction, and the initiation of chronic neuroinflammation. Several flavonoids have been shown to interfere with Aβ aggregation and toxicity [146,147].

Quercetin, in particular, has demonstrated the ability to inhibit Aβ fibrillogenesis and mitigate Aβ-induced cellular damage. Khan et al. [67] reported that quercetin effectively reduced Aβ fibril formation and counteracted Aβ-associated cell lysis and pro-inflammatory cascade activation. Similarly, a study by Ansari et al. [148] showed that quercetin inhibited Aβ-induced cytotoxicity and apoptotic cell death. Furthermore, Zhang et al. [149] discovered that quercetin increased the expression of apolipoprotein E (ApoE)—a protein involved in cerebral cholesterol metabolism and Aβ clearance—by inhibiting ApoE degradation in astrocytes.

Other flavonoids, such as luteolin, also exhibit anti-amyloidogenic effects. He et al. [150] demonstrated that luteolin not only inhibited Aβ aggregation but also promoted its enzymatic degradation, reduced neuronal apoptosis, and repaired mitochondrial damage. These effects were mediated through the inhibition of β-site amyloid precursor protein-cleaving enzyme 1 (BACE1), which is a key rate-limiting enzyme in Aβ generation, and the upregulation of insulin-degrading enzyme (IDE), a zinc-dependent metalloprotease responsible for Aβ clearance.

In addition to Aβ pathology, flavonoids may also modulate tau-related neurodegeneration. In AD, tau protein, which normally stabilizes microtubules, becomes hyperphosphorylated, leading to the formation of NFTs—a process that contributes to synaptic loss and neuronal death. Several flavonoids have been shown to interfere with tau phosphorylation, aggregation, and toxicity [151].

Rutin, a flavonoid glycoside, has been shown to inhibit tau aggregation and tau oligomer-induced cytotoxicity. In an in vitro study, Sun et al. [152] demonstrated that rutin reduced pro-inflammatory cytokine production, preserved neuronal morphology, and enhanced microglial clearance of extracellular tau oligomers. In vivo, rutin downregulated NF-κB signaling, increased protein phosphatase 2A (PP2A) activity (a major tau phosphatase), attenuated gliosis, and prevented synaptic loss in mouse models of tauopathy. These effects translated into significant improvements in cognitive performance, highlighting rutin’s potential to simultaneously target both Aβ and tau pathologies.

Another promising compound is isobavachalcone, which has been shown to inhibit tau aggregation and disassemble pre-formed tau fibrils in vitro. Xiao et al. [153] demonstrated that isobavachalcone directly interacts with tau protein and reduces its hyperphosphorylation by modulating the activities of glycogen synthase kinase 3β (GSK3β) and PP2A. Additionally, it conferred neuroprotection against tau-induced apoptosis, acting through mitochondria- and endoplasmic reticulum–mediated pathways. These findings support isobavachalcone as a candidate for further investigation in AD therapy.

Apigenin also shows potential anti-tau effects. In a study by Alsadat et al. [154], apigenin significantly reduced tau hyperphosphorylation in the hippocampus of rat models, further supporting its neuroprotective potential.

Taken together, these findings suggest that flavonoids provide a multifaceted neuroprotective approach in AD. By targeting Aβ aggregation, tau hyperphosphorylation, oxidative stress, and neuroinflammation, flavonoids act on several core pathological mechanisms simultaneously. Importantly, flavonoids such as quercetin, luteolin, and rutin have also been shown to improve cognitive function in animal models of AD, likely through the modulation of these interconnected pathways [140,144,152].

The ability of flavonoids to cross the BBB, combined with their favorable safety profiles, underscores their promise as both preventive and therapeutic agents for AD [139,141,143,152].

### 5.3. Impact on Cognitive Function and Memory

Flavonoids have been extensively studied for their potential neuroprotective effects in both AD and mild cognitive impairment (MCI). Evidence from preclinical and clinical studies suggests that flavonoids may offer cognitive benefits by targeting multiple pathological mechanisms, including oxidative stress, neuroinflammation, and amyloid and tau pathology [155,156].

In a randomized clinical trial, You et al. [157] demonstrated that 12 weeks of supplementation with *Cosmos caudatus*—a vegetable rich in flavonoids such as catechin, quercetin, proanthocyanidins, and rutin—resulted in improvements in global cognition, mood, tension, and oxidative stress in older adults with MCI. Notably, the mean percentage change in serum GSH levels was significantly higher in the supplementation group compared to placebo, indicating enhanced antioxidant defense.

Viña et al. [158] investigated the effect of genistein in individuals with prodromal AD and reported significant improvements in two neurocognitive tests, with a trend toward improvement in others. Imaging analyses showed that genistein-treated patients exhibited no increase in Aβ uptake in the anterior cingulate gyrus, in contrast to placebo-treated individuals, suggesting a potential role in slowing AD progression.

In a study by Cheatham et al. [159], older adults with MCI who consumed a flavonoid-rich lyophilized wild blueberry (*Vaccinium angustifolium*) powder supplement showed improved processing speed, as measured using the Cambridge Neuropsychological Test Automated Battery (CANTAB). After 6 months, performance levels in the treatment group approximated those of cognitively healthy individuals.

Morillas-Ruiz et al. [160] examined the effects of a polyphenol-rich antioxidant beverage, containing apple (*Malus pumila*), lemon (*Citrus limon*), and green tea (*Camellia sinensis*) extracts with added vitamins B and C, on plasma homocysteine (tHcy) levels in both healthy individuals and early-to-moderate AD patients. The beverage attenuated the rise in tHcy levels compared to placebo, which may be beneficial given the neurotoxic properties of elevated homocysteine.

Rosli et al. [161] conducted a randomized controlled trial to evaluate the effects of TP 3-in-1™, a polyphenol-rich tropical fruit juice containing pomegranate (*Punica granatum*) concentrate, guava (*Psidium guajava*), and roselle (*Hibiscus sabdariffa*) extract, on cognitive function and metabolomic profiles in middle-aged women with signs of MCI. After 10 weeks of supplementation, significant improvements were observed in verbal learning (RAVLT immediate recall) and processing speed (CTMT Trail 4) in the intervention group. The metabolomics analysis revealed increased urinary excretion of thyroxine and 3-methyladenine—compounds associated with neuroprotective mechanisms such as TTR stabilization and autophagy activation. These findings suggest that anthocyanin-rich tropical fruit juice may contribute to cognitive improvement through both functional and biochemical pathways.

Finally, do Rosario et al. [162] conducted a randomized, double-blind, placebo-controlled clinical trial in older adults with MCI who consumed a high-anthocyanin fruit juice (201 mg/day) for 8 weeks. The juice was prepared from a blend of blueberry (*Vaccinium angustifolium*), blackcurrant (*Ribes nigrum*), elderberry (*Sambucus nigra*), strawberry (*Fragaria ananassa*), and plums (*Prunus domestica* and *Prunus salicina*)—all fruits rich in anthocyanins. The intervention group showed a significant reduction in serum TNF-α compared to both the low-dose and placebo groups. This finding supports the potential of high-dose anthocyanin intake to mitigate neuroinflammation associated with cognitive decline.

These findings are summarized in Table 2, which outlines selected clinical and preclinical studies demonstrating the cognitive and biochemical effects of flavonoid-rich interventions in populations with cognitive impairment or AD.

Despite these promising clinical findings, it is important to recognize that many existing trials are limited by small sample sizes, short treatment durations, and substantial heterogeneity in study designs, cognitive assessment tools, and biomarker endpoints. These methodological differences complicate the comparison of results across studies and limit the generalizability of conclusions. Therefore, future research should prioritize large-scale, long-term, and methodologically standardized clinical trials to better establish the therapeutic potential of flavonoids in AD.

## 6. Potential for Combination Therapies—Synergistic Effects with Existing Alzheimer’s Disease Treatments

Current pharmacological treatment of AD relies on pro-cognitive agents, primarily acetylcholinesterase inhibitors (e.g., donepezil, rivastigmine) and NMDA receptor antagonists (e.g., memantine). However, these agents offer only symptomatic relief and limited disease-modifying potential. Recent research has explored the possibility of combining these drugs with flavonoids to enhance therapeutic efficacy through multimodal mechanisms [146,163,164].

In a rat model of scopolamine-induced amnesia, co-administration of donepezil and quercetin resulted in superior cognitive outcomes compared to monotherapies. Behavioral assessments using the Morris water maze, passive avoidance test, and elevated plus maze showed improvements in escape latency, transition latency, and transfer latency. Biochemical analyses revealed elevated GSH levels, reduced lipid peroxidation (LPO), decreased acetylcholinesterase (AChE) activity, and lower amyloid-β_1–42_ concentrations in the combination group. These findings suggest that the multitargeted action of quercetin may enhance the neuroprotective profile of donepezil [165].

Additional studies have confirmed the synergistic effects of donepezil when combined with flavonoids such as quercetin and kaempferol, derived from Diplazium esculentum (DE). This combination not only enhances AChE inhibition but also shows synergistic suppression of butyrylcholinesterase (BChE), another enzyme involved in AD pathology. Moreover, quercetin has demonstrated additive inhibitory effects on β-secretase 1 (BACE1) when used alongside donepezil. These synergistic interactions may allow for dose reduction of standard pharmacotherapy, potentially minimizing adverse effects, toxicity, and drug–drug interactions while maintaining or enhancing therapeutic outcomes [166].

## 7. Limitations and Future Research Directions

### 7.1. Current Research Gaps and Challenges

Despite promising preclinical evidence, several limitations hinder the clinical translation of flavonoid-based strategies for AD. One of the key challenges lies in the discrepancy between preclinical and clinical outcomes. Much of the current data originate from in vitro studies or animal models, which, although valuable, do not fully capture the complexity of human AD pathology. Rodent models, for instance, often lack the full spectrum of genetic heterogeneity, environmental influences, and comorbidities that characterize human disease. As a result, flavonoids that demonstrate efficacy in reducing neuroinflammation or amyloid accumulation in animal studies may yield inconclusive or inconsistent results in human trials, leading to potential overestimation of their therapeutic potential [167].

Another major limitation is the short duration of most clinical trials investigating flavonoids in AD. These trials often span only a few weeks or months, which is insufficient to evaluate long-term outcomes in a progressive, chronic neurodegenerative disorder such as AD. The delayed onset of neuroprotective effects and the slow rate of cognitive decline in early disease stages necessitate longitudinal studies to determine true efficacy. Furthermore, many trials are characterized by small sample sizes and limited demographic diversity, which reduces the statistical power and generalizability of findings. Differences in age, sex, ethnicity, and stage of cognitive impairment may all influence response to flavonoid-based interventions but are often insufficiently addressed [167,168,169].

A third critical challenge involves the bioavailability of flavonoids—that is, their ability to be absorbed, distributed, metabolized, and reach target tissues in the brain. Many flavonoids exhibit poor oral bioavailability due to low gastrointestinal absorption, rapid hepatic metabolism, and quick systemic elimination, which collectively limit their therapeutic potential when consumed in standard dietary forms [170,171]. Moreover, inter-individual variability—arising from differences in genetics, diet, and particularly gut microbiota composition—can significantly affect flavonoid metabolism and efficacy, posing further challenges in establishing consistent dosing regimens.

To address these issues, novel delivery strategies are under investigation, including nanoparticle carriers, liposomal formulations, and encapsulation techniques, aimed at enhancing flavonoid stability and brain penetration. Preliminary results from preclinical models are encouraging, but such approaches remain largely experimental and require validation in well-designed human clinical trials before they can be adopted in therapeutic protocols [172,173].

### 7.2. Future Directions in Flavonoid Research for Alzheimer’s Disease

To address the current limitations associated with flavonoid-based interventions for AD, future research should prioritize several key areas, including personalized therapeutic strategies, long-term clinical trials in diverse populations, advanced delivery technologies, identification of the most effective flavonoid compounds, biomarker discovery, and exploration of synergistic effects with conventional AD treatments.

Optimizing the therapeutic potential of flavonoids in AD will require a better understanding of how individual genetic, environmental, and lifestyle factors influence their efficacy. Emerging research suggests that personalized interventions—informed by genetic profiles, gut microbiota composition, and molecular biomarkers—could enhance the responsiveness to flavonoid-based therapies. Tailoring supplementation to the individual patient may improve consistency in clinical outcomes and allow for precision nutrition approaches in neurodegenerative disease management [174].

In addition, future studies should aim to identify and compare the effects of specific flavonoids, such as quercetin, luteolin, and apigenin, alone or in combination, to determine which compounds are most effective in targeting core AD mechanisms, including amyloid aggregation, tau pathology, oxidative stress, and neuroinflammation. Long-term clinical trials involving diverse and well-characterized populations, including variation in age, ethnicity, genetic background, and disease severity, are essential to establish dose–response relationships, the optimal duration of supplementation, and the clinical relevance of cognitive improvements [175].

Improving the bioavailability and CNS delivery of flavonoids remains a critical objective. Continued research into nanoparticles, liposomal carriers, and encapsulation technologies is warranted, as these delivery systems can enhance gastrointestinal absorption, protect against rapid metabolism, and promote effective brain penetration. Future strategies may also focus on targeted delivery to regions of the brain particularly affected in AD, such as the hippocampus and cerebral cortex, to further improve therapeutic efficacy [176].

Finally, the identification of robust biomarkers capable of predicting or monitoring responses to flavonoid supplementation is essential for advancing clinical translation. These may include molecular markers of neuroinflammation (e.g., TNF-α, IL-6), Aβ or tau levels, oxidative stress indicators, or performance-based cognitive assessments. Establishing such markers will allow researchers to stratify participants more effectively, monitor treatment effects, and refine clinical guidelines for flavonoid use in AD [177].

**Figure 2:**
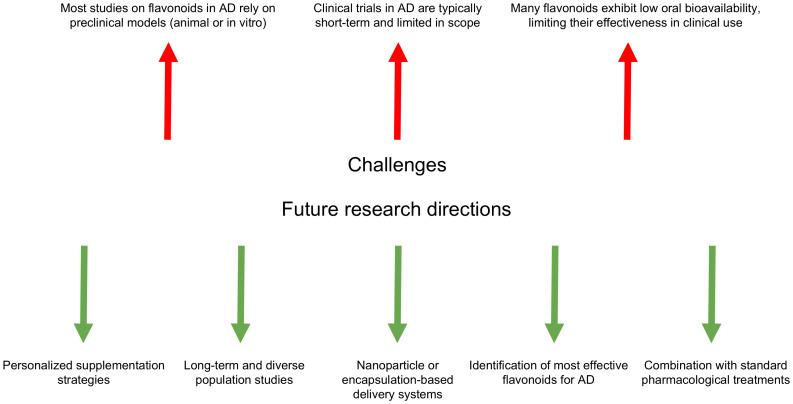
Key challenges and future research directions in flavonoid-based therapies for Alzheimer’s disease (AD).

These key challenges and proposed research directions are summarized in Figure 2, which illustrates the current limitations in flavonoid-based therapies for AD and highlights the strategic areas requiring further investigation.

## 8. Conclusions

Flavonoids represent a diverse group of naturally occurring polyphenolic compounds with substantial, though still not fully realized, therapeutic potential. Their multifaceted biological activity, particularly their antioxidant and anti-inflammatory properties, positions them as promising candidates for the prevention and treatment of numerous diseases, including AD. Increasing evidence from preclinical and early clinical studies suggests that flavonoids can modulate several key pathological mechanisms of AD, including oxidative stress, neuroinflammation, Aβ accumulation, and tau pathology.

However, the current body of clinical evidence remains limited by short study durations, small and demographically narrow cohorts, and insufficient data on long-term cognitive outcomes. These limitations hinder the broad translation of flavonoid-based interventions into routine clinical practice. To overcome these challenges, future research should prioritize the individualization of flavonoid therapy, long-term and population-diverse clinical trials, and the development of advanced delivery systems to enhance bioavailability and central nervous system penetration.

Moreover, the exploration of synergistic effects between flavonoids and existing pharmacological treatments may further amplify therapeutic efficacy. By addressing these critical gaps, future research will pave the way toward a more effective and clinically applicable role for flavonoids in the prevention and management of AD.

## Figures and Tables

**Table 1:** Major inflammatory markers involved in Alzheimer’s disease (AD) pathophysiology and their roles in neuroinflammation.

Inflammatory Marker	Role in AD Pathophysiology	Reference
α1-ACT	Alpha-1-antichymotrypsin is overexpressed in the AD brain. It promotes abnormal tau protein phosphorylation, leading to neurite loss and apoptosis, and contributes to amyloid-β (Aβ) aggregation.	[48]
CXCL-10	C-X-C motif chemokine ligand 10 recruits immune cells (T cells, monocytes) to the brain, promoting chronic neuroinflammation and accelerating neuronal damage. It is upregulated in AD and linked with glial activation.	[49]
Homocysteine	Elevated homocysteine levels cause oxidative stress, mitochondrial dysfunction, and inflammatory activation. It also impairs DNA repair, sensitizes neurons to Aβ toxicity, and is associated with tau pathology.	[50]
hs-CRP	High-sensitivity C-reactive protein increases blood–brain barrier (BBB) permeability and reflects systemic inflammation. Its elevated levels are associated with AD-related neuroinflammatory processes.	[51]
IFN-γ	Interferon-gamma enhances microglial uptake of Aβ, induces glial expression of MHC class II molecules, and may influence tau pathology. It plays a complex dual role in modulating immune activity in AD.	[52]
IL-1β	Interleukin-1 beta is a potent pro-inflammatory cytokine released by microglia. It increases BBB permeability, promotes Aβ accumulation, induces tau hyperphosphorylation, and contributes to synaptic dysfunction.	[28]
IL-6	Interleukin-6 triggers tau hyperphosphorylation via cdk5/p35 dysregulation, promotes chronic inflammation and astrocyte activation, and impairs synaptic plasticity linked to cognitive decline.	[33]
IL-8	Interleukin-8 recruits activated microglia to damaged brain areas and mediates neuron–glia interactions, amplifying local neuroinflammation and tissue damage.	[53]
TNF-α	Tumor necrosis factor-alpha promotes oxidative stress, microglial and astrocyte activation, apoptosis, and Aβ aggregation. It contributes to chronic synaptic dysfunction and neurodegeneration.	[33]

**Table 2:** Effects of selected flavonoids on cognitive function and biomarkers in preclinical and clinical studies.

Flavonoid/Source	Observed Effects	Reference
*Cosmos caudatus* (rich in catechin, quercetin, proanthocyanidins, rutin)	Improved global cognition, mood, and reduced oxidative stress; increased serum glutathione (GSH)	[157]
Genistein	Improved performance in neurocognitive tests; reduced amyloid-β (Aβ) deposition in the anterior cingulate gyrus	[158]
Wild blueberry (*Vaccinium angustifolium*) powder (flavonoid-rich, lyophilized)	Enhanced processing speed in older adults with MCI (CANTAB)	[159]
Antioxidant beverage (apple (*Malus pumila*), lemon (*Citrus limon*), green tea (*Camellia sinensis*) + vitamins B and C))	Attenuated increase in serum homocysteine (tHcy) in healthy and Alzheimer’s disease (AD) participants	[160]
Polyphenol-rich TP 3-in-1™ tropical fruit juice (pomegranate (*Punica granatum*), guava (*Psidium guajava*), and roselle (*Hibiscus sabdariffa*))	Improvements in verbal learning and processing speed; increased urinary thyroxine and 3-methyladenine levels associated with neuroprotection	[161]
Anthocyanin-rich fruit juice (blueberry (*Vaccinium angustifolium*), blackcurrant (*Ribes nigrum*), elderberry (*Sambucus nigra*), strawberry (*Fragaria ananassa*), plums (*Prunus domestica* and *Prunus salicina*))	Significant reduction in serum tumor necrosis factor-alpha (TNF-α), suggesting attenuation of neuroinflammatory processes relevant to AD pathology	[162]

## Data Availability

No new data were created or analyzed in this study. Data sharing is not applicable to this article.
